# Voltammetric Sensors Using Chemically Active Electrode Materials

**DOI:** 10.6028/jres.093.129

**Published:** 1988-06-01

**Authors:** Calvin O. Huber

**Affiliations:** Department of Chemistry, University of Wisconsin-Milwaukee, Milwaukee, WI 54301

Voltammetric analytical techniques have ordinarily used relatively inert conducting materials in order to maximize the range of available applied potential and to enhance selectivity of the electrode reactions by control of applied potentials. A number of investigators have recently been examining modification of electrode surfaces in order to improve selectivity and sensitivity which are essential components to enhanced accuracy at trace levels. These modifications include bonded chemical functionalities, adsorption, polymers, use of electrode paste vehicle solubility, enzyme attachment and others. In the work reported here the use of several chemically active electrode materials is reported.

In flow-through voltammetric devices, such as for HPLC and FIA detectors, the chemical history of the electrode surface is relatively easily managed so that sometimes chemically active electrode materials can offer advantages. Further, these onstream voltammetric configurations offer the advantages of controlled convection, electrode surface history, and ease of detector design. In this laboratory some recent studies exemplifying such an approach have involved nickel/nickel oxide [1–3], copper/copper oxide and silver iodide [4] as working electrode materials.

Nickel electrodes have allowed the smooth oxidation of hydroxyl and amine as well as more easily oxidized organic functional groups. The typical electrode conditions include 0.1 *M* sodium hydroxide as electrolyte. Often 0.1 m*M* nickel sulfate is added to the electrolyte to enhance long-term activity to slower reacting analytes. Typically a flow rate of 1 mL/min and a sample injection volume of 25 µL is used. The anodic current is controlled by redox reaction rate of the electrodes higher oxide lattice sites with the analyte molecule. This allows for low concentration level determinations of sugars, alcohols, glycols, amino acids, proteins, nucleic acids and nucleic acid constituents. Although the carrier stream is alkaline, acidic samples can be readily accommodated using the flow injection technique. The low-pH sample plug first produces a cathodic component to the signal as part of the electrode oxide layer is reduced, but as the pH increases with the passing of the low-pH segment a fresh, high-activity oxide layer is produced which oxidizes the end of the sample plug. Samples with pH as low as two can be accommodated. Layers of nickel oxide adsorbed on metals other than nickel yielded similar reactions to those using a nickel substrate.

Several proteins have been determined at concentrations as low as 1 mg/L [3]. Sensitivity is enhanced by increased temperature. Side- and end-hydroxyl and amino groups as well as sulfhydryl groups are oxidized, thus the technique is general for proteins, not limited to sulfide or bisulfide as for most electroanalytical methods, or to aromaticity as in UV-absorbance methods. Denaturation of human serum albumin in 0.1 *M* NaOH was followed by the technique. The results obtained correlate with a model based on random coil chain length control of denaturation rates interrupted by disulfide bond breakage delays.

A series of alcohols and glycols was studied. The anodic reaction rates, i.e., anodic amperometric sensitivities, could be correlated to the electron withdrawing nature of the moiety bonded to the primary reactant methylene-ol (–CH_2_OH) of the analyte. Rate constants vary by more than an order of magnitude from isopropanol to 1,2-propanediol. The linear range was more than two orders of magnitude, extending to lower detection limits of less than one micromolar.

The nickel electrode work has been extended to HPLC detection for amino acids by post-column mixing of 0.2 *M* sodium hydroxide at a T connection with a 0.1 mm × 1.3 m mixing tube [2]. As predicted by hydrodynamic theory, no significant band broadening was observed.

Consideration of Pourbaix diagrams indicates that cathodic pretreatment of a nickel electrode can generate a temporary high pH near the electrode surface. On stepping to anodic potentials a temporary anodic current for analytes should then be observed. Applying this approach resulted in the determination of ethanol in a pH 7 carrier solution with a linear response, a sensitivity of 3.3 µA/m*M* and a lower detection limit of 0.8 m*M.* This relatively high limit was primarily due to high background current.

Analytes subject to anodic determination at nickel can also be determined using a copper anode. Conditions are similar to those for nickel except that the applied potentials must be 200 mV more positive than for nickel. The anodic reaction rate constants with copper are typically somewhat greater than with nickel, however the applied potential results in increased background noise so that copper offers no signal-to-noise advantage over nickel.

When the background electrolyte contains nickel as suspended nickel hydroxide as described above, copper, nickel, cobalt, silver, platinum, and gold electrodes yield similar analytical currents. These results indicate that suspended nickel hydroxide adsorbs to the metal electrode surfaces and essentially converts them to nickel oxide electrodes.

Silver iodide in its room-temperature crystal form is sufficiently conducting so that it can carry the currents necessary for analytical amperometry. The electrode material is contacted using silver epoxy. The cathodic electrode mechanism involved generation of iodide at the silver:silver iodide interface yielding a current proportional to the rate of oxidation of iodide ions at the silver iodide:solution interface. Hypochlorous acid at sub-parts-per-million level thus yields cathodic currents for pH 6 solutions whereas pH 3 must be used for cathodic amperometry of monochloramine [4]. Accordingly a direct, linear response analytical technique for concentrations from more than 5 mg chlorine per liter down to about 10 µg chlorine per liter was developed for either monochloramine or the sum of hypochlorous acid plus monochloramine. The concentrations determined were sufficiently low to allow determination of the rate constant for monochloramine formation under realistic water treatment concentration, pH, and ionic strength conditions. The rate constant obtained was 3.2 × 10^6^ L/mol/s, in agreement with earlier reported values extrapolated from higher concentrations and less moderate pH solutions.

## References

[1] Hui BS, Huber CO (1982). Anal Chim Acta.

[2] Kafil JB, Huber CO (1985). Anal Chim Acta.

[3] Yuan CJ, Huber CO (1985). Anal Chem.

[4] Morrison TN, Huber CO, McKnight D (1987). Amperometric Measurement of Chlorine at a Pulsed Silver Iodide Electrode, The Chemical Quality of Water and the Hydrologic Cycle.

